# How Valuable Is the RT-qPCR of Pituitary-Specific Transcription Factors for Identifying Pituitary Neuroendocrine Tumor Subtypes According to the New WHO 2017 Criteria?

**DOI:** 10.3390/cancers11121990

**Published:** 2019-12-11

**Authors:** María Eugenia Torregrosa-Quesada, Araceli García-Martínez, Sandra Silva-Ortega, Sebastián Martínez-López, Rosa Cámara, Carmen Fajardo, Cristina Lamas, Ignacio Aranda, Antonio Picó

**Affiliations:** 1 Biochemical Department, Hospital General Universitario de Alicante, 03010 Alicante, Spain; eugenia_torregrosa@hotmail.es; 2 Research Laboratory, Hospital General Universitario de Alicante -ISABIAL, 03010 Alicante, Spain; araceli86gm@gmail.com (A.G.-M.); sebastian.martinez@goumh.umh.es (S.M.-L.); 3 Pathology Department, Hospital General Universitario de Alicante, 03010 Alicante, Spain; ssilvaortega@yahoo.es (S.S.-O.); ignaranda@gmail.com (I.A.); 4 Endocrinology Department, Hospital Universitario Politécnico La Fe, 46026 Valencia, Spain; rosacamaragomez@gmail.com; 5 Endocrinology Department, Hospital Universitario La Ribera, Alzira, 46600 Valencia, Spain; fajardo_carmon@gva.es; 6 Endocrinology Department, Complejo Universitario de Albacete, 02006 Albacete, Spain; clamaso@sescam.jccm.es; 7 Endocrinology Department, Hospital General Universitario de Alicante -ISABIAL, Miguel Hernández University, 03010 Alicante, Spain

**Keywords:** pituitary-specific transcription factor genes, pituitary-specific transcription factor proteins, pituitary-specific hormone genes, pituitary neuroendocrine tumors, classification of pituitary tumors

## Abstract

The classification of pituitary neuroendocrine tumors (PitNETs) subtypes continues generating interest. In 2017, the World Health Organization (WHO) proposed considering the immunohistochemical (IHC) analysis of pituitary-specific transcription factors (TF) for their typification. The present study targeted the quantification of pituitary-specific TF (*TPIT*, *PIT-1*, *SF-1*, *GATA2*, *ESR1*) gene expression by RT-qPCR to overcome the shortcomings of IHC and to complement it. We analyzed 251 tumors from our collection of PitNETs and performed additional IHC studies in a subset of 56 samples to analyze the concordance between gene and protein expression of the TF. The molecular and IHC studies allowed us to significantly reduce the percentage of null cell tumors in our series, most of which were reclassified as gonadotroph tumors. The concordance between the molecular and the immunohistochemical studies was good for tumors coming from the corticotroph and Pit-1 lineages but worsened for the rest of the tumors. Indeed, the RT-qPCR helped to improve the typification of plurihormonal Pit-1 and unusual tumors. Overall, our results suggest that the RT-qPCR of pituitary-specific TF and hormone genes could help pathologists, endocrinologists, and neurosurgeons to improve the management of patients with pituitary tumors.

## 1. Introduction

The World Health Organization (WHO) Classification of Tumors of Endocrine Organs, in its fourth edition, bases the classification of pituitary tumors on specific transcription factors, namely, pituitary-specific positive transcription factor 1 (Pit-1), Tbox family member TPIT (Tpit), and steroidogenic factor 1 (SF-1), involved in the differentiation of anterior pituitary cells [1]. The classification recognizes seven main subtypes of pituitary tumors: somatotroph tumors (ST), lactotroph tumors (LT), thyrotroph tumors (TT)—all of Pit-1 lineage—corticotroph tumors (CT, Tpit lineage), gonadotroph tumors (GT, SF-1 lineage), null cell (NC) tumors (no lineage), and plurihormonal tumors (Pit-1 lineage or variable combination of other transcription factors).

Pituitary neuroendocrine tumors (PitNETs), as they have been recently named [2], have increased in frequency mainly as a result of the exponential increase in cranial imaging techniques. A meta-analysis of 33 articles based on autopsy and radiological data showed that the prevalence of PitNETs ranged from 1% to 40% in the imaging studies, with similar results in the postmortem studies. On the basis of these data, the authors estimated an overall prevalence of PitNETs of 16.7% in the general population [3]. More recently, a nationwide study showed a prevalence of pituitary tumors of 115.57/100,000, with LTs being the most prevalent subtype, followed by non-functioning pituitary adenomas (NFPAs). However, over the whole surveillance period (1952–2012), NFPAs were the most frequently diagnosed tumors [4].

NFPAs are PitNETs without signs or symptoms of hormone hypersecretion, but whose growth can produce local pressure, resulting in visual impairment, headache, vomiting, diplopia, or dizziness. Nowadays, these tumors are known as silent pituitary tumors or silent PitNETs (sPitNETs). They are more invasive and recur more frequently than the functioning ones [5]. Indeed, the 2017 WHO classification of pituitary tumors highlights the importance of recognizing the specific tumor subtypes that show aggressive behavior, among which the sparsely granulated ST (frequently silent), the silent CT, and the plurihormonal Pit-1-positive tumor (formerly known as silent subtype 3 adenoma).

Given the increasing detection of PitNETs and their potential to produce clinical problems, an early and accurate diagnosis could have far-reaching benefits. Since a GT does not share the same characteristics and behavior as a CT, it is important to properly identify the tumor subtype. However, most of the studies published until now have considered sPitNETs as a single tumor type, with silent CTs the occasional exception.

In the era of precision medicine, targeted therapeutic approaches require identifying tumors as precisely as possible. The measurement of specific transcription factors’ expression will enable a better classification of the different PitNET subtypes, facilitating a more accurate approach to them. However, the 2017 WHO classification, like previous ones, is still based on immunohistochemistry (IHC). However, IHC has several shortcomings. In fact, we have observed important variability in the IHC results among four pathology departments participating in the typification of a large series of PitNETS [6]; moreover, the technique showed limitations in the identification of NC and plurihormonal tumors. The evaluation of transcription factors will hopefully resolve this problem, but interobserver variability will persist. On the other hand, we have recently demonstrated that the RT-qPCR of pituitary-specific hormone genes complements IHC studies, reducing the percentage of NC and unusual plurihormonal (UPH) tumors [6,7].

Consequently, the present study aimed to typify a large series of PitNETs according to the gene expression profiling of transcription factors, following the 2017 WHO guidelines, and to calculate the concordance between IHC and the molecular analysis of transcription factors in a subset of fully studied tumors in a single center.

## 2. Results

### 2.1. Patients

**Figure 1:**
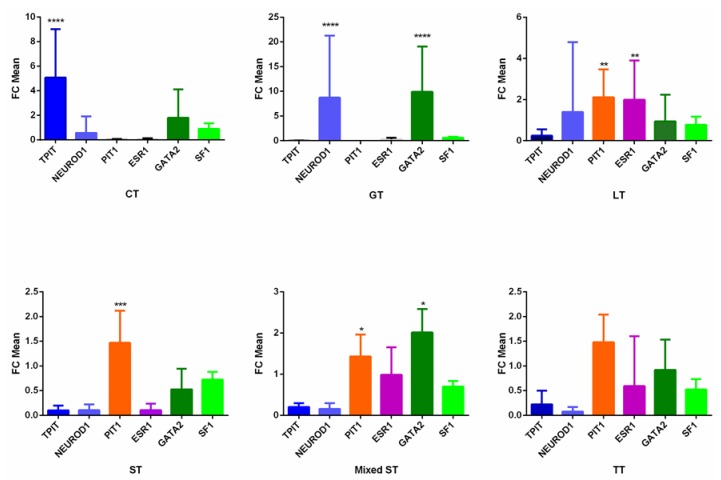
Mean fold change of transcription factors’ gene expression. FC, fold change; CT, corticotroph tumors; GT, gonadotroph tumors; LT, lactotroph tumors; ST, somatotroph tumors; TT, thyrotroph tumors; **** *p* < 0.0001; *** *p* < 0.001; ** *p* < 0.01; * *p* < 0.05, Kruskal–Wallis and Mann–Whitney tests.

In the present study, we investigated the gene expression of the pituitary-specific transcription factors in 251 PitNETs previously categorized in subtypes according to their pituitary-specific hormone gene profile (Figure 1). Of these, 168 PitNETs were silent tumors, 41 of which were NC tumors, and 71 were GTs. In addition, the same transcription factors were also studied by IHC in a subset of 56 tumors, 9 of which were NC tumors, and 19 were GTs. The remaining silent tumors included silent CTs, TTs, STs, LTs, and silent Pit-1 and UPH tumors.

The demographic, clinical, biochemical, immunohistochemical, and molecular characteristics of the 251 patients are shown in Supplemental Table S1. There was discordance between the molecular and the immunohistochemical identification of the PitNET subtypes in some patients. This discordance was attributed to the fact that the IHC studies were performed in different pathology departments, whereas the RT-qPCR was centralized in the Research Laboratory of the Alicante General University Hospital [6].

### 2.2. Identification and Frequency of PitNET Subtypes According to the Gene and Protein Expression of Pituitary-Specific Hormones

The pituitary-specific hormone genes used to identify the PitNET subtypes were *GH, FSH, LH, TSH*, *PRL*, *POMC*, *AVPR1B*, and *CRHR1***.** Table 1 shows the frequency of the different PitNET subtypes based on the expression of pituitary-specific hormone genes in the whole sample and on the IHC evaluation of pituitary hormone proteins in the subset. The most prevalent subtypes in the RT-qPCR study were GT and ST, followed by CT and NC. In the IHC study, the most prevalent subtypes also were GT and ST, followed by NC and CT.

### 2.3. Identification and Frequency of PitNET Subtypes According to the Gene and Protein Expression of Pituitary-Specific Transcription Factors

Identifying the dominant gene (251 tumors) and protein (56 tumors) expression of the pituitary-specific transcription factors allowed us to classify our collection of tumors as CTs, GTs, Pit-1 cell lineage tumors, UPH, and NC tumors. Besides the pituitary-specific transcription factor that defined the different PitNET subtypes, most tumors expressed all or some of the other transcription factors in different amounts (Table 2 and Figure 1). Remarkably, CTs expressed *TPIT* (Tpit) but also *GATA2*, while the GTs expressed *GATA2* and *NEUROD1.*

**Figure 2:**
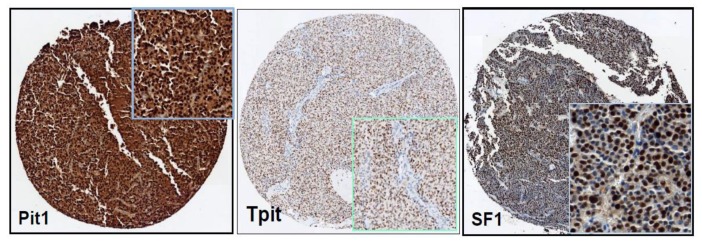
Representative images of the immunohistochemical expression of transcription factor.

Table 3. shows the transcription factor gene and protein expression profiles of the different PitNET subtypes of the subset, and Figure 2 shows representative immunohistochemical positive pictures. Moreover, the absolute and relative frequencies of the different PitNET subtypes according to their gene or protein expression of transcription factors are summarized in Table 4. The GTs were the most prevalent tumors, followed by tumors of the Pit-1 cell lineage and CTs, whereas the percentage of NC tumors was drastically reduced compared with the previous classification.

### 2.4. Concordance between Molecular and Immunohistochemical Identification of PitNET Subtypes in the Subset Sample

Table 5 shows the concordance in the identification of the PitNET subtypes between the gene (by RT-qPCR) and protein (by IHC) expression results of pituitary-specific transcription factors in the subset of 56 patients. Concordance was high between Tpit immunopositivity and *TPIT* expression and between Pit-1 immunopositivity and *PIT-1* expression. However, there was no concordance between the levels of expression of SF-1 by IHC and gene expression analysis. Instead, the gene expression of *GATA2* was concordant with the IHC expression of SF-1; therefore, we considered it useful in the identification of GTs.

## 3. Discussion

This is the first report characterizing a large series of PitNETs (n = 251) according to the gene expression of pituitary-specific transcription factors, following the recommendations of the recently released 2017 WHO classification of pituitary tumors. We demonstrated good concordance between the molecular and the immunohistochemical identification of the PitNET subtypes based on the expression of transcription factors in a subset of 56 patients. The strength of the study is that all the experiments were performed in a single center, in a surgical series of patients with complete clinical, biochemical, immunohistochemical, pathological, and molecular data. The most important limitation is that, occasionally, the molecular and immunohistochemical studies were performed in different areas of the same tumor. Moreover, the inclusion of samples with normal pituitary tissue hindered the interpretation of the molecular data in some cases. Therefore, samples that were suspected of being significantly contaminated were excluded from the study.

Tumors of endocrine organs produce hormones that may be associated to a specific clinical syndrome. This special characteristic has been used in their immunohistochemical identification with specific hormone antibodies [8]. However, the pituitary contains different cell lines responsible for producing specific hormones. Even though the 2004 WHO classification of pituitary tumors had the main advantage to consider the cytodifferentiation of the tumors, it failed to consider the transcription factors that target specific genes of these hormones. Indeed, the identification of these pituitary-specific transcription factors has allowed us to understand the plurihormonality shown by many PitNETs, providing new tools for a more specific and accurate typification of pituitary tumors, especially in the case of sPitNETs.

Nishioka et al. recently assessed the usefulness of the pituitary-specific transcription factors in the characterization of a large series of sPitNETs [9]. By studying 119 NC tumors by evaluating the expression of Pit-1, Tpit, SF-1, and estrogen receptor- α (ERα), the authors were able to reclassify 95% of the tumors as GTs, CTs, or Pit-1-derived tumors (LTs, STs, or TTs). On the basis of these and other results, the fourth edition of the WHO classification of endocrine tumors included a novel approach for classifying the PitNETs according to pituitary cell lineages [1]. This classification continues to be based on IHC for pituitary hormones, with the addition of the pituitary-specific transcription factors. However, the unavailability of well-trained staff and the difficulties in obtaining certain antibodies against the pituitary-specific transcription factors will make its worldwide implementation very difficult.

More and more frequently, the RT-qPCR of tumors is being incorporated into daily clinical practice. In the case of pituitary tumors, the gene expression quantification of the pituitary-specific transcription factors could give insights into the pathogenesis of these tumors and contribute to a better identification of different subtypes, especially the NC and plurihormonal ones. Indeed, previous results of our group addressed the complementary role of the RT-qPCR of pituitary-specific hormone genes and the IHC identification of PitNET subtypes. The percentages of NC and UPH tumors decreased from 12.7% to 7% and from 23.2% to 3.5%, respectively, in the first series [6] and from 17% to 12.5% and 17% to 9.8%, respectively, in the second one [7].

Similarly, the study of the gene expression of specific-pituitary transcription factors could complement their IHC analysis in the identification of PitNET subtypes according to the 2017 WHO classification of pituitary tumors.

Two corticotroph cell-specific regulators have been described: the basic helix–loop–helix transcription factor NEUROD1 (*NEUROD1)* and the pituitary-restricted transcription factor, Tpit (*TPIT)*. Both NEUROD1 and Tpit exert their transcriptional effects on the activation of *POMC* transcription through their interaction with Pitx1 [10,11]. Indeed, Tpit is coexpressed with *POMC* in both secreting and silent tumors of the corticotroph lineage and has not been found in the other types of PitNETs. Therefore, Tpit could be considered a marker of CTs [12]. In our series, *TPIT* (Tpit) was expressed in 86.36% of CTs, 23.07% of UPH tumors, and 4.88% of NC tumors, compared with 7.14% of TTs, 7.69% of LTs, 2.82% of GTs, and 0% of STs. In contrast, *NEUROD1* was expressed in all the PitNET subtypes, showing poor discriminatory utility.

Unexpectedly, the GTs of our series expressed levels of *SF-1* similar to those of other PitNET subtypes (Table 2, Figure 1). Therefore, this gene was not specific to the gonadotroph subtype in the present study. SF-1 is a transcription factor belonging to the steroid receptor superfamily. This transcription factor is expressed in human pituitary cells, where it regulates gonadotropin production, specifically, the gonadotropin β subunit [13]. Indeed, it has been reported that human TTs that produce the α but not the β subunit do not express *SF-1* [14]. In our series, only one TT (7.14%) expressed *SF-1*, and none produced the SF-1 protein. However, 36.62% of all GTs and 94.74% of the subset, expressed *SF-1* and the SF-1 protein, respectively. Since we used the hydrolysis probe with the best coverage, the discrepancy between gene and protein expressions could be attributed to other factors such as the possible influence of miRNAs that might affect protein expression. On the other hand, the stability of the SF-1 mRNA may have influenced its detection.

At the same time, 87.32% of GTs expressed *GATA2,* a zinc finger transcription factor involved in the development of gonadotroph cells. In a previous study, *GATA2* was detected by IHC and RT-PCR in 100% of the gonadotropin subunit-positive tumors [15]. Thus, this transcription factor could be considered a marker of GTs, underlining its utility in the correct diagnosis of the gonadotroph lineage of most of our previously identified GTs. Interestingly, 20.45% of the CTs of our series also expressed *GATA2*, and, contrarily, 74.65% of the GTs expressed *NEUROD1.* The co-expression of *GATA2* in CTs and *NEUROD1* in GTs suggests that some of our CTs and GTs could represent the cortico–gonadotroph tumor subtype previously suggested by Cooper et al. [16]. This subtype behaves as an entity with cellular characteristics of both gonadotroph and corticotroph tumors, expressing *NEUROD1* and SF-1 but little or no Tpit. All tumors are macroadenomas and clinically behave similarly to silent CTs. In addition, 50% of TTs also expressed *GATA2*. Besides its participation in the differentiation of gonadotrophs, *GATA2* also participates in the activation of the thyrotropin-subunit promoter [17,18]. Indeed, data have shown that *GATA2* is detected in the gonadotropin subunit-positive tumors and in most TTs [15]. These results suggest that the interaction between *GATA2* and Pit-1 could promote the differentiation of TTs.

As expected, STs, LTs, and TTs preferentially expressed the transcription factor Pit-1, both gene and protein. Pit-1 (*PIT-1*) is part of the homeobox family of proteins involved in cellular development. This transcription factor actively participates in the cell proliferation and hormonal activity of the pituitary, stimulating *GH* and *PRL* expression in both rodents and humans [19,20]. Moreover, it has been reported in TSH-, PRL-, and GH-secreting pituitary tumors. Unlike Pit-1 mRNA transcripts, which are found in all pituitary cells, the Pit-1 protein has only been described in thyrotroph, somatotroph, and lactotroph cells [21]. Its expression in tumors derived from other cell lineages, such as CTs, has been attributed to the presence of PRL, GH, or TSH-β mRNA-expressing cells in these tumors [22]. This could explain the concordance of only 0.825 (Kappa coefficient) between the molecular and the IHC determination of Pit-1 in our subset study. Therefore, the determination of *PIT-1* allowed us to reclassify five TTs and three LTs as other pituitary tumor subtypes. Conversely, some CTs and NC tumors were identified as Pit-1 cell lineage-dependent tumors.

Fifty-four percent of LTs and 4.22% of GTs expressed the estrogen receptor 1 gene (*ESR1)*, which was also found in 23.08% of UPH and 4.88% of NC tumors. However, some of our UPH tumors were *LH/FSH*-positive, and most NC tumors were finally re-classified as GTs. *ESR1* mediates the mitogenic effects of estrogens on pituitary cells. Two forms with different tissue patterns of expression exist: estrogen receptor (ER) α and ERβ. ERα has been limited to LTs and GTs, where it could modulate gonadal steroid estrogen (E_2_)-mediated gene expression [23]. In a previous study of 71 human PitNETs, the analysis of ER mRNA by RT-PCR showed significant expression of ERα in all LTs and in 61% of GTs [24]. Simultaneously, another study evaluating the mRNA expression of ERα and ERβ in 38 PitNETs found co-expression of both receptors in 60% of LTs and 29% of GTs. Therefore, the expression of *ESR1* could be helpful in identifying tumors especially of lactotroph but also of gonadotroph lineages.

Five plurihormonal tumors (four functioning and one silent) in the whole series (2%) (Table 1) expressed a combination of *GH, PRL*, or *TSH*, suggesting Pit-1 lineage classification. In all these cases (Table 2), the study of the *PIT-1* transcription factor confirmed the diagnosis. The Pit-1-positive tumor was previously called silent subtype 3 adenoma. Its prevalence was estimated at under 1% of all pituitary tumors, and its diagnosis was based on specific ultrastructural characteristics, including nuclear spheridia and prominent Golgi complexes in large cells with irregular nuclei [25]. Recently, a review of 29 PitNETs showed that this subtype included both functioning and silent tumors, mostly presenting TSH, GH, PRL, or ACTH immunoreactivity [26]. The subtype is considered very aggressive, prompting calls for its proper identification. The prevalence of plurihormonal Pit-1 tumors found in our molecular series is twice that published in the literature, suggesting that molecular studies could be more sensitive than IHC ones for detecting Pit-1 plurihormonal tumors. Indeed, our pathologist did not identify any plurihormonal Pit-1-positive tumors in the subset.

In our study, plurimorphous, plurihormonal tumors, which include two or more different lineages, were found in 5.6% and 12.5% (Table 4) of the tumors in the molecular and immunohistochemical analysis of transcription factors, respectively. In over 70% of the cases, the tumors were silent. The frequency of this subtype was similar when the tumors were identified according to the molecular expression of the transcription factors (5.6%, Table 4) and the molecular expression of the pituitary-specific hormones (5.2%, Table 1). Conversely, this frequency almost quadrupled in the immunohistochemical study (12.5%, Table 4) compared with the molecular one (3.6%, Table 1). UPH tumors are PitNETs derived from two or more independent cell lineages, so they express inexplicable IHC combinations by cytodifferentiation, and their etiology is not well known. Some authors have proposed that they could come from clonal expansions of uncommitted cells [27]. These unusual combinations have been scarcely documented, and most publications have been on isolated cases or double PitNETs. Moreover, the typification of these tumors has been based on the IHC study of pituitary-specific hormones [28,29,30,31,32]. Only Kageyama et al. [33] have described the IHC co-expression of NEUROD1, Tpit, and Pit-1 in a silent PitNET with positivity for ACTH and PRL. Our results suggest that this tumor subtype is more prevalent than initially thought. Whether it behaves as aggressively as the Pit-1 subtype remains to be clarified, but this question is beyond the scope of the present study.

Like Nishioka et al. [9], but using molecular studies of pituitary-specific transcription factors, we reclassified 80.5% (33/41, Supplemental Table S1) of the tumors previously deemed to be NC in the whole sample, reducing the frequency of this subtype from 16.3% to 3.2% (Table 1 and Table 4). Most of the NC tumors (31/33, 94%) were re-classified as GTs (Supplemental Table S1). Similar results were found in the subset with the IHC study: the frequency of NC tumors decreased from 16.1% to 3.6% (Table 1 and Table 4), while 77.77% (7/9, Supplemental Table S1) of tumors previously identified as NC PitNETs were classified as GTs. NC tumors were initially defined as chromophobic tumors without signs of pituitary-specific cell differentiation by IHC or specific characteristics in an electron microscopy study [34]. The 2017 WHO classification of endocrine tumors extended the definition of NC tumors to include the lack of evidence of pituitary-specific cell line differentiation depending on the IHC positivity of pituitary-specific transcription factors. Initially, NC tumors were thought to constitute up to 30% of sPitNETs, but more advanced IHC studies [35] and the study of pituitary-specific hormone genes [6,7] drastically reduced this estimate. The IHC study of transcription factors has further reduced the percentage of NC tumors from surgical series (< 5%) [9], as shown also in our study.

As other authors have found [9,35], most NC tumors in our study were re-classified as GTs. Gonadotroph and NC tumors share most characteristics in the pathological evaluation. Both are Periodic Acid Schiff (PAS)-negative chromophobic tumors and contain Golgi apparatus, endoplasmic reticulum, and small and spare secretory granules by electron microscopy. Indeed, several years ago, some studies demonstrated that NC tumors released β-LH or β-FSH in cell tissue cultures [36], and later studies detected their expression of α or β FSH mRNA or LH subunits by in situ hybridization [37,38]. Our previous studies of *FSH* and *LH* expression by RT-Qpcr [6,7] and the present analysis of transcription factors of gonadotroph lineage (*SF-1*, *GATA 2* and *ESR1)* are consistent with these results. Therefore, most of the tumors initially identified as NC tumors should be considered gonadotroph PitNETs.

Overall, there was acceptable concordance between the molecular and the IHC identification of PitNETs based on pituitary-specific transcription factors (Table 5). As in the molecular and IHC PitNET identification based on pituitary-specific hormone gene and protein expression [6,7], the concordance was good for CTs and Pit-1 tumors but worsened for GTs and especially for UPH and NC tumors. The 2017 WHO classification of endocrine tumors continues recommending the analysis of transcription factors and pituitary hormones by IHC. However, some antibodies to transcription factors, such as SF-1, are not readily available, and in addition, the IHC techniques require very well trained pathologists, restricting the analysis of transcription factors to a few pathology departments. Moreover, the protein expression of a specific gene is sometimes aborted, reducing the sensitivity of IHC in identifying the cellular origin of the tumor. Indeed, we recently reported that molecular analysis contributed to the typification of a cystic prolactinoma whose pathological study was negative for all pituitary hormones [39]. Therefore, we believe that the molecular determination of the pituitary-specific transcription factor and hormone genes significantly complements the IHC study, expanding the number of Pituitary Centers of Excellence that could benefit from a better typification of PitNETs.

## 4. Materials and Methods

To study the contribution of the analysis of gene expression of specific pituitary transcription factors to the typification of PitNET subtypes, we performed a prospective study of SF-1, GATA 2, TPIT, and Pit-1 in a large series of pituitary tumors previously characterized accordingly to their expression of pituitary-specific gene hormones. In addition, we also studied the protein expression of these transcription factors (except for GATA-2) in a subset of tumors to calculate the concordance between gene and protein expression.

### 4.1. Patients and Samples

We selected 251 tumors from our collection of PitNETs for which we had enough biological material to analyze the molecular expression or transcription factors. Anonymized clinical, immunohistochemical, and molecular data were collected from the Spanish Molecular Registry of PitNETs (REMAH) database, which is part of a Spanish multicenter project. This was a retrospective study in which the RNA quantity and quality were determined using the NanoDrop Spectrophotometer. In addition, RNA integrity was evaluated by the 4200 TapeStation system (Qiagen) in a subset of 96 samples, since we did not have enough material for many of the samples to perform this analysis in the whole series, due to the small size of this type of tumors. Briefly, 79.2% of the samples showed an RNA Integrity Number (RIN) higher than 6 compared to 15.6% of samples that showed an RIN lower than 6; 5.2% of samples could not be evaluated. The eventual bias introduced by a low-quality RNA was overcome by the use of samples that came from tissue frozen and stored in RNAlater (which prevents RNA degradation), the use of an optimized extraction kit which delivers optimal results in all downstream applications including RT-qPCR analysis, the normalization against two reference genes, a short amplicon size, and random hexamer priming.

### 4.2. Identification of PitNET Subtypes According to Their Clinical Behavior and the Expression of Pituitary-Specific Hormone Genes

Different PitNET subtypes were identified according to the criteria previously published by our group [7]. Briefly, functioning and silent PitNETs were determined depending on the presence of a recognizable endocrine syndrome, and different PitNET subtypes were identified according to the dominant pituitary-specific hormone gene expression.

The identification of tumor subtypes was based on the dominant gene (to define the concept of gene dominance in a specific PitNET subtype, we calculated the interquartile (p25–p75) expression of the specific genes for the corresponding, clinically identified subtype, i.e., the interquartile range of *GH* expression in clinically active naïve acromegalic patients) and protein expression of one or more hormones: (1) *POMC, AVPR1B*, and *CRHR1*/ACTH for CTs; (2) *GH/*GH for STs and *GH–PRL*/GH–PRL for ST mixed or mammosomatotroph tumors; (3) *PRL/*PRL for LTs; (4) *TSH/*TSH for TTs; (5) *FSH*–*LH/*FSH–LH for GTs. Finally, when there was dominant gene or protein expression for more than one pituitary-specific hormone, we classified the tumor as UPH, and when there was no expression of the studied genes/proteins, we considered the tumor as NC. We considered as diagnostic a fold change (by RT-qPCR) of a pituitary-specific hormone gene above p25 and an immunopositivity of a pituitary-specific hormone protein of more than 5% (by IHC).

### 4.3. Molecular and IHC Identification of PitNET Subtypes According to Transcription Factors

In all tumors of the whole sample, we analyzed the gene expression of the main transcription factors related to the three pituitary cell lineages: *TPIT*, *NEUROD1*, *PIT-1*, *ESR1*, *GATA2*, and *SF-1*. Moreover, in a subset of 56 tumors we simultaneously studied the protein expression of the corresponding transcription factors (Tpit, Pit-1, and SF-1) by IHC to calculate the concordance between molecular and immunohistochemical identification of PitNET subtypes.

The identification of tumor subtypes was based on the dominant gene and protein expression of one or more transcription factors: 1. *TPIT/*Tpit for CTs; 2. *PIT-1/*Pit-1 and *GATA2/ESR1* for tumors of the Pit-1 lineage; 3. *GATA2/SF-1/*SF-1 for GTs. When there was dominant gene or protein expression of more than one pituitary cell lineage, we classified the tumor as UPH, and when there was no expression of the studied genes/proteins, we considered the tumor as NC. GATA2, NEUROD1, and ESR1 were not quantified by IHC. We considered as diagnostic a fold change (by RT-qPCR) of a specific transcription factor gene above p25 and an immunopositivity of a specific transcription factor protein over 5% (by IHC).

### 4.4. Concordance between Molecular and Immunohistochemical Identification of PitNET Subtypes According to the Gene or Protein Expression of Transcription Factor

Once we identified the different PitNET subtypes according to the gene and protein expression of the transcription factors studied in the subset of 56 patients, we calculated the concordance between the molecular and the immunohistochemical identifications (kappa coefficient).

### 4.5. Discordant Cases

Once we molecularly identified all cases on the basis of transcription factors’ expression, we observed some discordance with respect to the pituitary-specific hormone gene identification. Specifically, 7 CTs were re-classified as GTs, UPH, and Pit-1; 5 GTs as NC and UPH; 4 STs as NC and UPH; 3 LTs as GTs and CTs; 5 TTs as UPH, GTs, NC, and CTs; 6 UPH as GTs, NC, and Pit-1; and 40 NCs as GTs, UPH, and Pit-1. All these cases are summarized in Supplemental Table S1.

### 4.6. RNA Extraction and cDNA Synthesis

All molecular studies were centralized in the Alicante General University Hospital Research Institute (ISABIAL) laboratory.

All samples were preserved immediately after surgery in RNAlater solution at 4 °C for 24 h and then stored at −20 °C. The biological samples were disintegrated in the TissueLyser (Qiagen, Hilden, Germany). We used the AllPrep DNA–RNA-Protein kit (Qiagen) for manual RNA extraction (treated with DNase) and measured RNA concentration and purity in the Nanodrop spectrophotometer (Thermo Scientific, Waltham, MA, USA).

For each retrotranscription reaction, we used 2 μg of RNA in a total volume of 20 μL, employing the High-Capacity cDNA Reverse Transcription kit (Applied Biosystems) which uses the reverse transcriptase MultiScribe™. The conditions for the thermal cycler were: 10 min at 25 °C, 120 min at 37 °C, and 5 min at 85 °C and, finally, hold at 4 °C.

### 4.7. Quantitative Real-Time Polymerase Chain Reaction (RT-qPCR)

We performed RT-qPCR following the manufacturer’s instructions for the Quant Studio 12K (Life Technologies, CA, USA), using assays based on hydrolysis probes. We selected the following assays: *TPIT* (Hs00193027_m1 and Hs.PT.58.26777525), *NEUROD1* (Hs01922995_s1 and Hs.PT.58.38524795), *PIT-1* (Hs00230821_m1 and Hs.PT.58.3315301), *GATA2* (Hs00231119_m1 and Hs.PT.58.961996), *ESR1* (Hs01046816_m1 and Hs.PT.58.14846478) and *SF-1* (Hs01124206_m1 and Hs.PT.58.4383285)*TPITPIT-1* As endogenous controls we selected *PGK1* (Hs00943178_g1 and Hs.PT.58.27790011) and *TBP* (Hs00427620_m1 and Hs.PT.58.19489510). The total volume per reaction was 10 µL: 5 µL master mix, 1 µL RT-PCR product, 0.5 µL hydrolysis probe, and 3.5 µL nuclease-free water. The RT-qPCR conditions were: 2 min at 50 °C, 20 s at 95 °C (polymerase activation), and 40 cycles of PCR (1 s at 95 °C plus 20 s at 60 °C).

A pool of RNA from nine normal pituitary samples obtained from autopsies served as a calibrator. All samples were analyzed in duplicate. The relative differences in gene expression were expressed as fold change and were obtained with the 2^−ΔΔCt^ method (SDS software, Applied Biosystems).

### 4.8. IHC Techniques

Fifty-six tumors previously diagnosed according to the 2004 WHO classification criteria were studied. Tissue microarrays (TMAs) were constructed with the selected cases. To do that, two 1 mm paraffin cylinders of each tumor were sampled to build a block using a matrix tissue device (Beecher instruments). Each block included 20 cases plus 2 controls. The TMAs were exposed to a panel of antibodies against the following pituitary cell lineage transcription factors: Pit1 (ThermoFisher PA5-59662), SF1 (abcam ab217317), and Tpit (abcam ab243028). The quantification of the immunostaining was performed by three observers using a Multivision microscope. The results were expressed as percentage of immunostained cells.

### 4.9. Ethics

The study complies with the Declaration of Helsinki and other applicable laws and received the approval of the local ethics committee (CEIm reference number: PI2018/127, date: 08 October 2018, Alicante General University Hospital). None of the donors came from a vulnerable population, and all donors or relatives freely provided written informed consent.

### 4.10. Statistical Analysis

Qualitative variables (including PitNET subtypes) were expressed as absolute and relative frequencies. Participants’ age and tumor diameter were expressed as mean ± standard deviation. We used the Shapiro–Wilk test to investigate normality in the distribution of the molecular variables (fold change of transcription factor genes). To compare qualitative and quantitative variables, we used the Kruskal–Wallis and the Mann–Whitney U test. We calculated the Cohen’s kappa coefficient to measure concordance between the IHC and the molecular identification of the different subtypes of PitNET (κ = 1 representing complete concordance, and κ ≤ 0 null concordance). We calculated the interquartile range and mean fold change to elaborate the figures; *p* values of less than 0.05 were considered statistically significant. Statistical analysis was performed with the SPSS 24.0 software (IBM Software; Miguel Hernandez University, Alicante, Spain).

## 5. Conclusions

The RT-qPCR of pituitary-specific transcription factor genes allows a better typification of PitNETs, significantly reducing the percentage of NC tumors, which were mostly reclassified as gonadotroph tumors. Moreover, it complements the IHC study in the proper typification of plurihormonal Pit-1 and unusual tumors and gives insights into the pathogenesis of PitNETs.

On the basis of our results, we consider that whenever IHC studies of pituitary-specific transcription factors are not available, RT-qPCR of pituitary-specific transcription factor genes and hormones could help pathologists, endocrinologists, and neurosurgeons to improve the management of patients with pituitary tumors.

## Figures and Tables

**Table 1:** Frequency of PitNET subtypes identified according to the gene (a) (whole series) and protein (b) (subset) expression of pituitary-specific hormones.

PitNET	(a)	(b)
	Molecular n (%) Hormones	IHC n (%) Hormones
ST	46 (18.3)	12 (21.4)
F-ST	44 (95.6)	11 (91.7)
S-ST	2 (4.3)	1 (8.3)
LT	17 (6.8)	4 (7.1)
F-LT	8 (47.1)	4 (100)
S-LT	9 (52.9)	0 (0.0)
TT	14 (5.6)	3 (5.4)
F-TT	3 (21.4)	1 (33.3)
S-TT	11 (78.6)	2 (66.7)
PH Pit-1	5 (2.0)	0 (0.0)
F-PH Pit-1	4 (80)	
S-PH Pit-1	1 (20)	
CT	44 (17.5)	7 (12.5)
F-CT	19 (43.2)	2 (28.6)
S-CT	25 (56.8)	5 (71.4)
GT	71 (28.2)	19 (33.9)
NC	41 (16.3)	9 (16.1)
UPH	13 (5.2)	2 (3.6)
UPH-F	5 (38.5)	0 (0.0)
UPH-S	8 (61.5)	2 (100)
Total	251 (100)	56 (100)

PitNET, pituitary neuroendocrine tumor; IHC, immunohistochemistry; F, functioning; S, silent; PH, plurihormonal; NC, null cell tumor; UPH, unusual plurihormonal tumor.

**Table 2:** Molecular expression of transcription factors in the whole sample.

Molecular Subtype (n)	*PIT-1*	*TPIT*	*SF-1*	*GATA2*	*NEUROD1*	*ESR1*
	*mRNA n (%)*	*mRNA n (%)*	*mRNA n (%)*	*mRNA n (%)*	*mRNA n (%)*	*mRNA n (%)*
**ST (50)**	48 (96)	0 (0)	5 (10)	6 (12)	8 (16)	4 (8)
**GT (71)**	0 (0)	2 (2.82)	26 (36.62)	62 (87.32)	53 (74.65)	3 (4.22)
**CT (44)**	3 (6.82)	38 (86.36)	7 (15.90)	9 (20.45)	18 (40.90)	0 (0)
**TT (14)**	10 (71.43)	1 (7.14)	1 (7.14)	7 (50)	5 (35.71)	1 (7.14)
**LT (13)**	10 (76.92)	1 (7.69)	1 (7.69)	5 (38.46)	2 (15.38)	7 (53.85)
**UPH (13)**	5 (38.46)	3 (23.07)	3 (23.07)	7 (53.85)	8 (61.54)	3 (23.08)
**PH-Pit-1 (5)**	5 (100)	0 (0)	1 (20)	1 (20)	2 (40)	0 (0)
**NC (41)**	4 (9.76)	2 (4.88)	7 (17.07)	38 (92.68)	25 (60.97)	2 (4.88)

ST, somatotroph tumor; GT, gonadotroph tumor; CT, corticotroph tumour; TT, thyrotroph tumor; LT, lactotroph tumor; UPH, unusual plurihormonal tumor; NC, null cell tumor.

**Table 3:** IHC and molecular expression of transcription factors in the subset of samples.

Subtype IHC (n)	Pit-1		Tpit		SF-1		*GATA2*
	*IHC n (%)*	*mRNA n (%)*	*IHC n (%)*	*mRNA n (%)*	*IHC n (%)*	*mRNA n (%)*	*mRNA n (%)*
**ST (12)**	12 (100)	12 (100)	0	0	2 (16.66)	1 (8.33)	4 (33.33)
**GT (19)**	2 (10.53)	0	1 (5.26)	0	18 (94.74)	2 (10.53)	17 (89.47)
**CT (7)**	0	0	7 (100)	7 (100)	0	1 (14.28)	3 (42.86)
**TT (3)**	2 (66.66)	2 (66.66)	0	0	0	0	2 (66.66)
**LT (4)**	4 (100)	3 (75)	0	0	0	1 (25)	1 (25)
**UPH (2)**	1 (50)	0	0	0	1 (50)	0	2 (100)
**NC (9)**	2 (22.22)	0	0	0	9 (100)	1 (11.11)	8 (88.88)

IHC, immunohistochemistry; ST, somatotroph tumors; GT, gonadotroph tumors; CT, corticotroph tumors; TT, thyrotroph tumors; LT, lactotroph tumors; UPH, unusual plurihormonal tumors; NC, null cell tumors.

**Table 4:** Frequency of PitNET subtypes identified according to the molecular and IHC expression of transcription factors in the whole and subset series, respectively.

PitNET	Molecular n (%) TF	IHC n (%) TF
Pit-1	79 (31.3)	17 (30.4)
Pit-1-F	64 (81.0)	14 (82.3)
Pit-1-S	15 (19.0)	3 (17.7)
CT	39 (15.5)	7 (12.5)
F-CT	18 (46.1)	2 (28.6)
S-CT	21 (53.9)	5 (71.4)
GT	111 (44.0)	23 (41.1)
NC	8 (3.2)	2 (3.6)
UPH	14 (5.6)	7 (12.5)
UPH-F	3 (21.4)	2 (28.6)
UPH-S	11 (78.6)	5 (71.4)
Total	251 (100)	56 (100)

PitNET, pituitary neuroendocrine tumor; TF, transcription factor; F, functioning; S, silent; CT, corticotroph tumor; GT, gonadotroph tumor; NC, null cell tumor; UPH, unusual plurihormonal tumor.

**Table 5:** Concordance between molecular and IHC identification of transcription factors.

Subtypes	IHC N	Molecular TF N	Kappa	*p*-Value
CT	7	6	0.913	<0.001
GT	23	30	0.612	<0.001
Pit-1	17	15	0.825	<0.001
UPH	7	3	0.135	0.262
NC	2	2	−0.037	0.782

IHC, immunohistochemistry; TF, transcription factor; CT, corticotroph tumor; GT, gonadotroph tumor; UPH, unusual plurihormonal tumor; NC, null cell tumor.

## Supplementary Materials

The following are available online at https://www.mdpi.com/2072-6694/11/12/1990/s1, Table S1: Demographic, clinical, biochemical, immunohistochemical, and molecular characteristics of the study series.
